# Utilization of community health care centers and family doctor contracts services among community residents: a community‐based analysis in Shenzhen, China

**DOI:** 10.1186/s12875-021-01444-6

**Published:** 2021-05-24

**Authors:** Qingming Zheng, Lu Shi, Tiantian Pang, Willie Leung

**Affiliations:** 1 Luohu Center for Disease Control and Prevention, Shenzhen, Guangdong China; 2 College of Public Health and Human Sciences, School of Social and Behavioral Health Sciences, Oregon State University, 2520 SW Campus Way, Corvallis, OR 97331 USA; 3 College of Public Health, University of South Florida, Tampa, FL USA; 4 College of Public Health and Human Sciences, School of Biological and Population Health Sciences, Oregon State University, Corvallis, OR USA

**Keywords:** Family doctor contract services, Family doctor, Andersen’s Behavioral Model, Shenzhen, Awareness, Chronic disease, Community health care center

## Abstract

**Background:**

Family doctor contract services (FDCS) began in China in 2016. Shenzhen, one of the most developed cities in China, also implemented a family doctor (FD) policy in 2017. The objectives of this study were to identify the impact of awareness of FDCS and signing service contracts with FDs on utilization of community health care centers (CHCs).

**Methods:**

Cross-sectional secondary data based on residents living in Luohu district was used for analysis. Descriptive analysis was conducted to identify utilization of CHCs by awareness of FDCS and signing service contracts with FDs, respectively. Linear probability models (LPM) were used to determine the association of utilization of CHCs with awareness of FDCS and signing service contracts with FDs, respectively.

**Results:**

Among 1205 adults included in the analysis, 27 % of the participants knew about the FDCS, 5 % signed with FD, and 20 % had chronic disease. Both awareness of the FDCS and signing service contracts with FDs significantly increased the probability of using CHCs as a first choice.

**Conclusions:**

This study provided evidence that both awareness of FDCS and signing service contracts with FDs had a positive impact on utilization of primary health care services at the community level. More interventions to improve awareness of FDCS are needed to increase the utilization of primary health care services.

**Supplementary Information:**

The online version contains supplementary material available at 10.1186/s12875-021-01444-6.

## Introduction

Even though the primary health care system was introduced to China in the 1980s, little attention was paid to establishing an effective primary health care system, such as training primary health care doctors. Due to the economic reforms in the early 1980s, the Chinese government reduced public health care expenses and the market-oriented health care reforms caused an increased concentration of health care resources at higher-level health care facilities [1, 2]. The neglect of the primary health care system by the government caused discontent among the public [3]. Thus, in recent years, the Chinese government has been trying to strengthen the ability of the primary health care system to provide health care services [1].

These efforts to strengthen primary care are exemplified by the rise of community health care centers (CHCs), the leading health care providers in the primary health care system in urban China [1]. Primary health care services provided by CHCs include 14 basic public health services, such as chronic disease management and vaccination [1, 4]. Increasing subsidies to primary health care institutions from $2.8 billion in 2008 to $20.3 billion in 2015 was one of the most critical health care reform policies related to CHCs [1]. The policy lead to an increased number of CHCs from 24,000 to 2008 to 35,000 in 2018 [5].

With the implementation of primary health care, the concept of primary physicians also known as family doctors (FDs) or general practitioners (GPs) was introduced to Chinese society. Primary physicians are the primary health care professionals in CHCs. To strengthen the primary health care system within the community and CHCs, the Chinese government introduced family doctor contract services (FDCS) in 2009 and promoted these services to the general public in 2016 [6]. FDCS are health care services provided by signing service contract with FDs in the CHCs [7]. Signing service contract with FDs to use FDCS is voluntary. FDs working as a primary health care workforce and providing FDCS may be able to increase the utilization of CHCs.

According to previous study in Shanghai, signing service contracts with FDs and awareness of FDCS significantly increased the effectiveness of self-management in health [8]. Recently, more researchers have been interested in exploring factors associated with signing service contracts with FDs and awareness of FDCS [9, 10]. Other research has also suggested that chronic disease patients were more likely to sign service contracts with FDs [11]. Previous studies estimating the association between influencing factors and signing service contract with FDs, the relationship between influencing factors and awareness of FDCS, and satisfaction of FDCS have shown that FDCS played an important roles in health care services in communities [9, 12, 13]. However, most of these studies or surveys were conducted in Shanghai [8, 9, 11, 13, 14], Guangzhou [10], and Zhenjiang province [12], limited research regarding FDCS has been conducted in Shenzhen.

Shenzhen is a southeastern metropolis located in Guangdong, China, and China’s first Special Economic Zone (SEZ), where it attracts millions of migrants to work, live, and study [15]. Luohu, a district in Shenzhen, was the first district in Shenzhen and in China to introduce the concept of health care group to manage the health care institutions [15]. The health care group is operated by one independent legal entity and its sub-entities share responsibility for providing health care services [15]. Luohu health care group includes 6 district level hospitals, 23 CHCs, and 1 health care research center [16]. With the introduction of health care group, CHCs in Shenzhen can efficiently aggregate health care resource to support community health [15]. It is important to note that not all health care services can be covered by the city’s or province’s health care funding. But there will be out-of-pocket expenditures by Chinese citizen utilizing health care services, including CHCs. However, usually, the reimbursement rate of health care is lower comparing to other health care institutions when received health care services at CHCs [17]. Further, regardless of insurance status, Chinese citizens have access to their local CHCs for health care services [17]. According to the Shenzhen Family Doctor Service Administrative Measures, CHCs can promote FDCS with professional FD teams, including GPs, public health physicians, pharmacies, etc. Under the management of health care group, community residents have easy access to professional FDs and can use FDCS from CHCs [15]. Thus, it is profoundly importance to study the association of awareness of FDCS and signing service contracts with FDs with utilization of primary health care services in Shenzhen.

Andersen’s Behavioral Model of Health Services Use provides a conceptual framework to identify factors associated with health care use [18]. We adapted the model to identify factors that affect CHCs utilization. According to the model, the utilization of CHCs is determined by three types of factors: predisposing, enabling, and needs. Predisposing factors are demographic characteristics, such as age, gender, education, and marital status. Enabling factors can be defined as insurance status, such as the availability of insurance. Need factors are the awareness of FDCS and signing service contracts with FDs.

The purpose of this study is to identify the impact of awareness of FDCS and signing service contracts with FDs on the probability of community residents choosing CHCs as their most frequently visited health care institutions. We hypothesized that community residents would be more likely to choose CHCs as their primary site for health care services if they were aware of FDCS or had signed service contracts with FDs.

## Methods

### Data and study population

Data were collected as part of the China Community Diagnosis Survey. The China Community Diagnosis Survey is a community health diagnosis program operated by the Centers for Disease Control and Prevention (CDC) of Shenzhen to track community health care needs in the Dongmen community within the Luohu district of Shenzhen, China, in 2019. As of 2019, Luohu district included 1.03 million residents [19]. The diversity of the Dongmen community fully represents the residents of Luohu district, and partially represents the residents of Shenzhen. The objectives of the survey are to identify the community’s health care problems, and to improve evidence-based health care promotion. Participants were community residents who lived in the districts for more than 6 months. Multistage cluster sampling was used to identify the sample of 1,205 residents. Seven sub-communities in the Dongmen community, Luohu district were selected. From these seven sub-communities, twenty-five clusters were randomly selected based on the community grid. A total of 925 households were interviewed face-to-face by trained investigators. If the selected household was not available, a nearby neighbor was chosen as a replacement, but the replacement rate was strictly controlled to within 5 % of the total sample. A total of 2,086 individuals were interviewed, of which 1,205 residents between 18 and 59 years old were included in the study. All participants provided written informed consent before participated in the survey. This secondary data analysis was approved by the IRB of the corresponding author’s institution.

## Variables

To identify the impact of awareness of FDCS and signing service contracts with FDs on utilization of CHCs, all variables utilized in this secondary analysis were from the China Community Diagnosis Survey in Luohu district. The outcome variable of utilization of CHCs as first choice was identified by the question “Which medical institutions do you frequently visit?” The response options included (1) private clinics, (2) CHCs, (3) secondary hospitals, and (4) tertiary hospitals. The response was recoded as binary variable into (1) CHCs and (2) Others. The independent variable of awareness of FDCS was based on the question, “Have you ever heard about FDCS?” The response options included (1) Yes and (2) No. The other independent variable of signing service contracts with FDs was measured by the question, “Did you sign a service contracts with a FD?”. The response options included (1) Yes and (2) No.

Socio-demographic information included: *hukou* status (Shenzhen *hukou*, non*-*Shenzhen *hukou*), age (18–29, 30–39, 40–49, 50–59), gender (male, female), marital status (Yes or No), education (no education or primary school, middle school, high school, or professional college or university), employment status (Yes or No), and insurance status (Yes or No). *Hukou* is a household registration system in China [20]. The *hukou* system identified individuals as rural or urban residents. Chronic disease status was measured with the following questions: “Do you have hypertension?” (Yes or No); “Do you have diabetes?” (Yes or No); “Do you have any following disease? Stroke, chronic bronchitis, chronic hepatitis B, hyperlipemia, cataract, osteoporosis, benign prostatic hyperplasia, cervical spondylopathy, chronic enteritis, chronic rhinitis, renal calculus, psychosis, benign tumors, malignant tumors, coronary disease, asthma, or other chronic disease?” (Yes or No). Participants’ responses were classified as binary variable of having chronic diseases, if they reported yes to any of the above questions and not having any chronic disease if they reported no to all of the above questions.

### Statistical analysis

The relationships between utilization of CHCs as first choice and (1) the awareness of FDCS and (2) likelihood of signing service contracts with a FD were estimated using a chi-squared test and linear probability models (LPM) via ordinary least squares (OLS), respectively. LPM coefficients, reported as percentage change, directly estimated the change in magnitude and direction of probabilities of usage of CHCs. All regression models were adjusted for socio-demographic information, including age, marital status, education, gender, employment status, insurance status, and chronic disease status. Bootstrapped standard error was estimated. Alpha levels were set at 0.05. All analyses were conducted using Stata/SE 16 (StataCorp LP, College Station, TX).

## Results

A total of 1,205 adults were included in the analysis. Table 1 presents the characteristics of the sample by awareness of FDCS and signing service contracts with FDs. Approximately 26.8 % of participants were aware of FDCS, but only 5.2 % of participants confirmed that they had signed service contracts with FDs. Approximately 55.7 % of participants who knew about FDCS selected CHCs as their first-choice health care institutions, compared to those who were not aware of the FDCS (*p* < 0.01). Approximately 61.9 % of participants who signed service contracts with FDs chose CHCs as their most frequent visited health care institutions, compared to those who did not sign service contracts with FDs (*p* < 0.01).

**Table 1:** Characteristics for utilization of CHCs by awareness of the FDCS and signing service contracts with FDs in Dongmen community, Luohu district, Shenzhen, China

Variable	Awareness of FDCS		χ2 P-value	Signing service contracts with FDs		χ2 *P*-value
	No, n (%)	Yes, n (%)		No, n (%)	Yes, n (%)	
Total, n	882 (73.20)	323 (26.80)		1142 (94.77)	63 (5.23)	
Utilization of CHCs						
Others	548 (62.13)	143 (44.27)	< 0.01	667 (58.41)	24 (38.1)	< 0.01
CHCs	334 (37.87)	180 (55.73)		475 (41.59)	39 (61.9)	
*Hukou* status						
Locals (Shenzhen *hukou*)	281 (31.86)	172 (53.25)	< 0.01	404 (35.38)	49 (77.78)	< 0.01
Migrants (non-Shenzhen *hukou*)	601 (68.14)	151 (46.75)		738 (64.62)	14 (22.22)	
Age						
18–29	218 (24.72)	51 (15.79)	< 0.01	262 (22.94)	7 (11.11)	< 0.01
30–39	253 (28.68)	121 (37.46)		356 (31.17)	18 (28.57)	
40–49	235 (26.64)	85 (26.32)		305 (26.71)	15 (23.81)	
50–59	176 (19.95)	66 (20.43)		219 (19.18)	23 (36.51)	
Gender						
Male	414 (46.94)	134 (41.49)	0.09	522 (45.71)	26 (41.27)	0.49
Female	468 (53.06)	189 (58.51)		620 (54.29)	37 (58.73)	
Marriage						
No	234 (26.53)	48 (14.86)	< 0.01	275 (24.08)	7 (11.11)	0.02
Yes	648 (73.47)	275 (85.14)		867 (75.92)	56 (88.89)	
Education						
No education or primary school	131 (14.85)	17 (5.26)	< 0.01	147 (12.87)	1 (1.59)	< 0.01
Middle school	261 (29.59)	64 (19.81)		314 (27.50)	11 (17.46)	
High school	245 (27.78)	99 (30.65)		321 (28.11)	23 (36.51)	
Professional college or university	245 (27.78)	143 (44.27)		360 (31.52)	28 (44.44)	
Employment status						
No	137 (15.53)	65 (20.12)	0.06	182 (15.94)	20 (31.75)	< 0.01
Yes	745 (84.47)	258 (79.88)		960 (84.06)	43 (68.25)	
Insurance status						
No	94 (10.66)	7 (2.17)	< 0.01	101 (8.84)	0 (0)	0.01
Yes	788 (89.34)	316 (97.83)		1041 (91.16)	63 (100)	
Chronic disease status						
No	729 (82.65)	240 (74.30)	< 0.01	934 (81.79)	35 (55.56)	< 0.01
Yes	153 (17.35)	83 (25.70)		208 (18.21)	28 (44.44)	

*Note*. Data from Community Diagnosis Questionnaire, Luohu, Shenzhen (2019)

*FD * Family doctor, *FDCS * family doctor contract services, *CHCs * community health care centers

The impact of awareness of FDCS on utilization of CHCs was estimated by LPM, as shown in Table 2. Our analysis found that participants who were aware of FDCS were 18.8 % points (*p* < 0.001) more likely to choose CHCs as their first choice of health care institutions compared to those who were not aware of FDCS. The analysis found that participants with insurance coverage were 18.4 % points (*p* < 0.001) more likely to choose CHCs as their first choice compared to uninsured participants. No statistically significant difference was found in other covariances. The analysis found a decreasing trend of selecting CHCs as first choice of health care institutions as education level increased. 

**Table 2:** Linear probability models examine association between utilization of CHCs and awareness of the FDCS in Dongmen community, Luohu district, Shenzhen, China

		Utilization of CHCs	
		Percentage point change (bootstrapped SE%)	*p*-value
Awareness of the FDCS			
No	Ref.		
Yes		18.806 (3.273)	< 0.001
*Hukou* status			
Locals (Shenzhen *hukou*)	Ref.		
Migrants (non-Shenzhen *hukou*)		5.164 (3.553)	0.146
Age			
18–29	Ref.		
30–39		4.869 (4.661)	0.296
40–49		7.492 (4.568)	0.101
50–59		3.906 (5.780)	0.499
Gender			
Male	Ref.		
Female		1.316 (3.071)	0.668
Marriage			
No	Ref.		
Yes		3.185 (4.100)	0.437
Education			
No education or primary school	Ref.		
Middle school		1.773 (5.150)	0.731
High school		-3.880 (5.559)	0.485
Professional college or university		-10.040 (5.847)	0.086
Employment status			
No	Ref.		
Yes		-1.106 (3.779)	0.770
Insurance status			
No			
Yes		18.428 (4.795)	< 0.001
Chronic disease status			
No	Ref.		
Yes		-4.573 (3.574)	0.201

*Note*. Data from Community Diagnosis Questionnaire, Luohu, Shenzhen (2019)

*FD* Family doctor, *FDCS* family doctor contract services, *CHCs* community health care centers

* *p* < 0.05, ** *p* < 0.01, *** *p* < 0.001

The relationship between signing service contracts with FDs and utilization of CHCs was estimated by LPM, as shown in Table 3. The results of LPM showed that participants who signed service contracts with FDs were 22.1 % points (*p* < 0.01) more likely to choose CHCs as their first choice of health care institutions compared to those did not sign service contracts with FDs. The analysis found that participants with insurance coverage were 20.3 % points (*p* < 0.001) more likely to choose CHCs as their first choice compared to uninsured participants.

**Table 3:** Linear probability models examine association between utilization of CHCs and signing service contracts with FDs in Dongmen community, Luohu district, Shenzhen, China

		Utilization of CHCs	
		Percentage point change (bootstrapped SE%)	*p*-value
Signing service contracts with FDs			
No	Ref.		
Yes		22.128 (6.752)	0.001
Hukou status			
Locals (Shenzhen *hukou*)	Ref.		
Migrants (non-Shenzhen *hukou*)		5.407 (3.718)	0.146
Age			
18–29	Ref.		
30–39		5.837 (4.611)	0.206
40–49		7.679 (4.515)	0.089
50–59		3.599 (5.723)	0.529
Gender			
Male	Ref.		
Female		2.194 (3.031)	0.469
Marriage			
No	Ref.		
Yes		4.959 (4.101)	0.227
Education			
No education or primary school	Ref.		
Middle school		2.810 (5.143)	0.585
High school		-1.592 (5.518)	0.773
Professional college or university		-6.571 (5.920)	0.267
Employment status			
No	Ref.		
Yes		-1.031 (3.787)	0.785
Insurance status			
No	Ref.		
Yes		20.293 (4.876)	< 0.001
Chronic disease status			
No	Ref.		
Yes		-4.605 (3.610)	0.202

*Note*. Data from Community Diagnosis Questionnaire, Luohu, Shenzhen (2019)

*FD * Family doctor, *FDCS* family doctor contract service, *CHCs* community health care centers

* *p* < 0.05, ** *p* < 0.01, *** *p* < 0.001

## Discussion

The results of this study indicate a positive impact of the FD policy implementation among the communities in Shenzhen. Individuals were more likely to choose CHCs as their first choice of health care institutions, if they were aware of FDCS or had signed service contracts with FDs. Even though individuals in the communities are free to visit any type of health care institutions, they were more willing to utilize CHCs compared to other health care institutions, which suggests that both awareness of FDCS and signing service contracts with FDs have a positive effect among residents in terms of the communities in utilizing CHCs.

The positive relationships between FDs and community’ residents showed that individuals living in Shenzhen were able to accept the FDCS, which was consistent with results from previous studies conducted in other cities [9, 10, 12, 14]. On the other hand, this also shows that there is still much room to improve the awareness rate of FDCS among residents in Shenzhen. The improving awareness rate about FDCS can have positive impact on utilization of CHCs.

With the publication of “Healthy China 2030” in 2016, it was suggested that FDCS should play an important role in achieving the goal of Healthy China 2030, such as improving population health by changing lifestyles [21]. As FDs were designed to serve community residents, FDs should become a supportive mechanism in primary health care. In 2016, the National Healthcare Security Administration released a new guideline to promote community residents’ willingness to visit and sign service contracts with FDs [22]. According to the guideline, in 2017, the service contracts signing rate with FDs should reach 30 % for the general population and 60 % for the population in special needs, such as elderlies, patients with chronic diseases. The guideline also suggested that by 2020, FDCS coverage should reach 100 % of the general population.

Compared to other cities, only 26.8 % of participants in Shenzhen were aware of FD services. Additionally, only 5.2 % of participants confirmed that they had signed service contracts with FDs in Shenzhen as of 2019. Compared to the contracts rate targets set by the National Healthcare Security Administration, which were 30 % by 2017 and 100 % by 2020, contracting with FDs in Shenzhen communities was far from meeting the target contracts rate [22]. Compared to Shenzhen, the signing rate was much higher in other comparable cities, such as the nearby cities of Guangzhou and Shanghai, with the contracts rate ranging from 14.4 to 65.3 % [8–10, 14, 23]. These differences have a number of possible explanations. First, the lower service contracts signing rate in Shenzhen may be explained by the high migration rate. According to the Shenzhen Statistical Yearbook, in 2019, only 35 % of the population in Shenzhen had Shenzhen *hukou* registration status [19]. Most of the population in Shenzhen were not permanent residents of the city, especially migrant workers who often moved to other cities in pursuit of other opportunities. The high migration rate may contribute to the low contracts rate with FDs, because the purpose and objective of FDs are to build up stable and consistent relationships with communities’ residents regarding health-related behaviors. Second, the short implementation time of FD policy may have led to a low service contracts signage rate with FDs. The FD policy was introduced at the end of 2017, in November. Most of the population might not have had a chance to become familiar with or be informed about the new policy of FDCS. Compared to Shanghai, Shenzhen also has a shorter history of FDCS. Shanghai was the first city to pilot FDCS in 2013 [7, 9]. Third, previous studies recruited study populations from CHCs, whereas our study population were recruited randomly from communities, which may better represent the usage of CHCs among the general population. Last, it is possible that residents in the community might have signed service contracts with FDs by their employers or insurance plans but unable to identify or confirm the exact FDs with whom they have signed.

An interesting finding was the great impact of education on choosing CHCs as first choice. Specifically, the probability of choosing CHCs as first choice decreased with education level. This may be largely explained by the trust of primary health care institutions and social media usage. Individuals with higher education level are more likely to absorb information from different mediums, such as social media [24]. It is possible that residents can received different perspectives of information from various sources, both in-person or online. One of the perspective could be residents do not trust primary health care providers [1]. Other perspective could be residents with higher education levels have more resources to become aware of the FDCS and FD policy implementation. However, this study cannot determine the reasons and teh causations fo the decreasing trends of residents with higher education selecting CHCs as their first choice. 

Even though the FD policy has only been implemented for a short period in Shenzhen, there is a need to further increase awareness of FDs and likelihood of signing service contracts with FDs. FDs should have the availability and the capacity to serve as health promoters in the communities. To serve as health promoters, FDs should play the following roles: (1) building relationships with local community members, (2) increasing personal medical skills, and (3) increasing the acknowledgement of the health promotion role of CHCs [15, 22]. The key concept is to build trust between FDs and community residents through providing home health care services to the community residents first. FDs cannot simply stay in the CHCs and wait for patients, instead, they should initiate contact with community residents. They can provide drop-in visits for some residents, such as individuals with chronic disease, individuals with disability, and the elderly. Social interaction is important to attract individuals to sign service contracts with FDs. Also, it is important to build up a consistent training environment. The distrust of primary care was mainly caused by the health care quality provided by GPs [1]. FDs should be trained consistently via either self-training or government-provided health care training. The impact of the FDCS will increase with the constant professional development of FDs. To expand the publicity of FD, social media should be used to advertise FDCS, such as using Weibo, Wechat, television, and newspaper. Diverse methods of health education and advertising should be implemented to promote the efficiency and advantages of FDCS.

 Due to the special health care group operation in Shenzhen, to the authors knowledge, this is one of the few studies identifying the impacts of awareness of FDCS on utilization of CHCs, and signing service contracts with FDs on utilization of CHCs. But this study is not without limitations. First, this study was a cross-sectional study with a focus on one district in Shenzhen. The data was collected through a self-reporting survey, self-reporting, recall, and social bias may exist, such as the variable of chronic disease status. Also, this survey was targeting on the health care needs in the community. It might not be able to capture all perspectives of FDSC and FD in Shenzhen. Lastly, this study only concentrated on Dongmen community, Luohu District, which may limit the generalizability of the study. Even though Dongmen community can represent in Luohu district but it may only partially represent Shenzhen. To fully investigate the contracted rate in Shenzhen, further studies need to be conducted on a larger scale.

## Conclusions

Even though the rates of FDCS awareness and signing service contracts with FDs were relatively low in Luohu, Shenzhen, they both have positive impacts on the utilization of CHCs. The results of this study confirm the positive impact of FDs on utilization of CHCs, aligned with previous studies. With the environment of policy implementation in primary health care, populations are gradually transforming their health care seeking behaviors. Community residents are more willing to utilize primary health care institutions, such as CHCs, as their first choice of health care institutes. Ultimately, it is also important for local government and society to make joint efforts to improve the medical services in the primary health care system, such as expanding public acceptance, improving health care quality for primary health care, and building up trust between community residents and FDs.

## Supplementary Information

**Additional file 1**

## Data Availability

The datasets generated during the current study are not publicly available due to the ownership belong of this dataset belong to Luohu Center for Disease Control and Prevention, Shenzhen, Guangdong, China. The datasets used and/or analyzed during the current study available from the Luohu Center for Disease Control and Prevention, Shenzhen, Guangdong, China on reasonable request.

## Abbreviations

CHCs: Community health care centers
CDC: Centers for Disease Control and Prevention
FDCS: Family doctor contract services
FD: Family doctor
GP: General physician
LPM: Linear probability models
OLS: Ordinary least squares
SEZ: Special Economic Zone
