# Supplementary material for: Utilization of community health care centers and family doctor contracts services among community residents: a community‐based analysis in Shenzhen, China
Source: BMC Fam Pract. 2021 May 24;22:100. doi: 10.1186/s12875-021-01444-6 (PMC8146992; doi:10.1186/s12875-021-01444-6)
Supplement: Supplementary file 1 — Additional file 1 [file 12875_2021_1444_MOESM1_ESM.docx]

**Community Diagnosis Questionnaire**

| **Part One: Household survey question** | |
| --- | --- |
| code | Question |
| f1 | In the past 6 months, what is the number of individuals living within your family? (Including relatives, nannies, etc., but not including family members that that have been away for more the 6 months) |
|  | Type Number |
| f2 | Within the number of individuals living with you, how many of them have Shenzhen *Hukou*? |
|  | Type Number |
| f3 | Which of the followings best describe your housing? |
|  | 1) Self-build house; 2) Commercial build house; 3) Renting or Room share |
| f4 | How would you rate your environmental hygiene in your residential area? |
|  | 1) Good; 2) Average; 3) Bad |
| f5 | How often do you open your window to increase air flow? |
|  | 1) 0-2 times/wk (poor); 2) 3-5 times/wk (fair); 3) 5 time or more/wk (good) |
| f6 | Within a day, does your bedroom have natural sunlight? |
|  | 1) Yes; 2) No |
| f7 | From April to October, are there any mosquitoes or flies inside your house? |
|  | 1) No (good); 2) One or two (fair); 3) more than two (poor) |
| f8 | Does your home have window screen installed? |
|  | 1) Yes; 2) No |
| f9 | Do you disinfect the eating utensils often? |
|  | 1) Yes; 2) No |
| f10 | Do you have any of the following commonly used medicine in your home? (multiple options) |
| f10_1 | No commonly used medicine at all |
|  | 1) Yes |
| f10_2 | Cold related medicine |
|  | 1) Yes |
| f10_3 | Trauma related medicine, such as hydrogen peroxide, iodine tincture |
|  | 1) Yes |
| f10_4 | Medical related instruments, such as bandage, tweezers |
|  | 1) Yes |
| f10_5 | Stomach related medicine |
|  | 1) Yes |
| f10_6 | Dermatologic related medicine, such as Mupirocin ointment |
|  | 1) Yes |
| f10_7 | Measurement instruments, such as sphygmomanometer |
|  | 1) Yes |
| f10_8 | Other medicine |
|  | 1) Yes |
| f10_8_text | Type the medicine name |
|  | Type answer |
| f11 | Within the past year, how much did your family spend on medical services and other medical utilities? (Including all spending but not including supplemental food and supplemental equipment) |
|  | Type Number |
| f12 | How much was pay out-of-pocket? (Not including supplemental food and supplemental equipment) |
|  | Type Number |
| f13 | Have you household ever controlled the amount of salt consumed (voluntarily control everyday food items' salt levels)? |
|  | 1) Yes; 2) No |
| f14 | Have you household ever controlled the amount of oil used during cooking (voluntarily control the amount of oil used every day)? |
|  | 1) Yes; 2) No |
| f15 | In a typically day, how many people eat dinner together in your household (including non-household members)? |
|  | Type Number |
|  |  |
| **Part Two: Household member survey question** | |
| code | Question |
| a1 | Who response to the following survey questionnaires? |
|  | 1) Self 2) Others |
| a2 | The reasons for others responding the survey questionnaires. |
|  | 1) Not home at the present of the survey 2) Too young 3) Low education 4) Not sound minded 5) Refuse to answer |
| a3 | Age |
|  | Type Number |
| a4 | Gender |
|  | 1) Male; 2) Female |
| a5 | What is the member's ethnicity |
|  | 1) Han; 2) Non-han |
| a6 | Types of *Houku* (household registration) |
|  | 1) Shenzhen *hukou* 2) Non-Shenzhen *hukou* |
| a7 | Marital status |
|  | 1) Single; 2) married; 3) divorce; 4) Widowed 5) Others text (a7_5_text) |
| a7_5_text | Marital status: other |
|  | Type answer |
| a8 | Education levels |
|  | 1) No-education; 2) Elementary school; 3)Middle school; 4) High school; 5) Special/professional high school; 6) College; 7) Master or above |
| a9 | Occupation |
|  | 1) Government official; 2) Scientific and technical staff; 3) Medical personnel; 4) Blue collar; 5) Sevicer; 6) Teacher 7) Financial industry worker; 8) Student; 9) Retired; 10) Unemployed; 11) Housewife; 12) Farmer; 13) Military; 14) Small business owner; 15) Private business owner/enterpriser 16) Self-employed 17) Other_text (a9_17_text) |
| a9_17_text | Occupation: other |
|  | Type answer |
| a10 | What types of insurances do you currently enroll in? (multiple options) |
| a10_1 | Insurance types: basic social insurance, including Urban Resident Basic Medical Insurance (URBMI) and Urban Employee Basic Medical Insurance (UEBMI) |
|  | 1) Yes |
| a10_2 | Insurance types: Shenzhen worker insurance. |
|  | 1) Yes |
| a10_3 | Insurance types: New Cooperative Medical Scheme (NCMS) |
|  | 1) Yes |
| a10_4 | Insurance types: commercial insurance. |
|  | 1) Yes |
| a10_5 | Insurance types: no insurance |
|  | 1) Yes |
| a10_6 | Insurance types: cooperative insurance |
|  | 1) Yes |
| a10_7 | Insurance types : non-adult insurance |
|  | 1) Yes |
| a10_8 | Insurance types: other insurance |
|  | 1) Yes |
| a10_8_text | Insurance types: other insurance text |
|  | Type answer |
| a11 | Do you have annual physical examination? |
|  | 1) Yes; 2) No |
| a12 | Do you have hypertension? (if the answer is "No", jump to a14) |
|  | 1) Yes; 2) No |
| a13 | Where did you receive your diagnosis of hypertension? |
|  | 1) Private clinic; 2) Community health care center; 3) District level general hospital; 4) City level general hospital; 5) District level or city level special hospital; 6) Province level hospital; 7) others |
| a13_7_text | Type the other health care institution name |
|  | Type answer |
| a14 | Do you have diabetes? (if the answer is "No", jump to a16) |
|  | 1) Yes; 2) No |
| a15 | Where did you receive your diagnosis of diabetes? |
|  | 1) Private clinic; 2) Community health care center; 3) District level general hospital; 4) City level general hospital; 5) District level or city level special hospital; 6) Province level hospital; 7) others |
| a15_7_text | Type the other health care institution name |
|  | Type answer |
| a16 | Have you ever diagnosis with any other disease or medical conditions? |
| a16_1 | No other disease |
|  | 1) Yes |
| a16_2 | Stroke |
|  | 1) Yes |
| a16_3 | Chronic bronchitis |
|  | 1) Yes |
| a16_4 | Chronic hepatitis B |
|  | 1) Yes |
| a16_5 | Hyperlipemia |
|  | 1) Yes |
| a16_6 | Cataract |
|  | 1) Yes |
| a16_7 | Osteoporosis |
|  | 1) Yes |
| a16_8 | Prostatauxe |
|  | 1) Yes |
| a16_9 | Cervical spondylopathy |
|  | 1) Yes |
| a16_10 | Chronic enteritis |
|  | 1) Yes |
| a16_11 | Chronic rhinitis |
|  | 1) Yes |
| a16_12 | Renal calculus |
|  | 1) Yes |
| a16_13 | Psychosis |
|  | 1) Yes |
| a16_14 | Benign tumor |
|  | 1) Yes |
| a16_15 | Malignant tumors |
|  | 1) Yes |
| a16_16 | Coronary disease |
|  | 1) Yes |
| a16_17 | Asthma |
|  | 1) Yes |
| a16_18_text | Other disease |
|  | Type answer |
| a17 | Two weeks before this interview, did you feel sick or diagnosis with acute or chronic disease? (If the answer is "No", jump to a29) |
|  | 1) Yes; 2) No |
| a18 | What are the symptoms? (multiple options) |
| a18_1 | Fever |
|  | 1) Yes |
| a18_2 | Dizzy |
|  | 1) Yes |
| a18_3 | Panic |
|  | 1) Yes |
| a18_4 | Cough |
|  | 1) Yes |
| a18_5 | Diarrhea |
|  | 1) Yes |
| a18_6 | Abdominal pain |
|  | 1) Yes |
| a18_7 | Rash |
|  | 1) Yes |
| a18_8 | Trauma |
|  | 1) Yes |
| a18_9 | Other |
|  | 1) Yes |
| a18_9_text | Other disease |
|  | Type answer |
| a20 | Was the disease or medical conditions you receive diagnosed with severe? |
|  | 1) Yes; 2) No |
| a21 | When did the symptoms first appear? |
|  | 1) New symptoms appear within 2 weeks; 2) Acute illness/symptoms appear 2 weeks ago and continues until these 2 weeks; 3) Chronic disease for two weeks |
| a22 | Two weeks before this interview, how many days did the symptoms lasted? |
|  | Type Number |
| a23 | Two weeks before this interview, did you receive time off or sick leave due to disease/medical conditions? |
|  | 1) Yes; 2) No |
| a24 | After the diagnosis, did you receive any treatments? If so, what was the treatments? |
|  | 1) No treatment; 2) Yes, self-treatment (jump to a29); 3. Yes, go to the professional medical institutions; 4) self-treatment and go to the professional medical institutions; 5) other |
| a24_text | Other treatments |
|  | Type answer |
| a25 | Within the past 2 weeks, which types of medical institutions did you receive your diagnosis? |
|  | 1) Private clinic; 2) Community health care center; 3) District level general hospital; 4) City level general hospital; 5) District level or city level special hospital; 6) Province level hospital; 7) others |
| a25_text | Type the other health care institution name |
|  | Type answer |
| a26 | What was the reason(s) you visited the previous mention medical institutions? (multiple options) |
| a26_1 | Proximity to the home |
|  | 1) Yes |
| a26_2 | Reasonable cost |
|  | 1) Yes |
| a26_3 | Skilled personnel |
|  | 1) Yes |
| a26_4 | Quality medical equipment |
|  | 1) Yes |
| a26_5 | Abundant medicine types |
|  | 1) Yes |
| a26_6 | Excellent customer services |
|  | 1) Yes |
| a26_7 | Assigned relationship to the health care institutions |
|  | 1) Yes |
| a26_8 | Familiarity with personnel |
|  | 1) Yes |
| a26_9 | Trust in certain physicians |
|  | 1) Yes |
| a26_9_text | Other reasons |
|  | Type answer |
| a27 | After meeting with the doctor, did you purchase the prescribed medicines at non-visit medical institutions and/or pharmacies? |
|  | 1) Yes; 2) No |
| a28 | What was the primary reason(s) you did not receive treatments for your diagnosis? |
|  | 1) Feels good; 2) Financial burden; 3) No time; 4) Inconvenient; 5) Medical services bad; 6) Not worth to treat; 7) Other |
| a28_text | Other reasons |
|  | Type answer |
| a29 | Within the past year were you hospitalized due to illness? (If the answer is "No", jump to b1) |
|  | 1) Yes; 2) No |
| a30 | What was the reason(s) for the hospitalization? |
|  | 1) Illness; 2) Trauma or poison; 3) Recovery; 4) birth control; 5) Get birth; 6) other |
| a30_text | Other reasons |
|  | Type answer |
| a31 | What was the diagnosis for hospitalization? |
|  | Type answer |
| a32 | Within the past year, how many times were you hospitalized due to the disease/medical conditions? |
|  | Type Number |
| a33 | Your recent hospitalizations were at which of the following medical institutions? |
|  | 1) District level general hospital; 2) City level general hospital; 3) District level or city level special hospital; 4) Province level hospital; 5) others |
| a33_text | Other medical institutions |
|  | Type answer |
| a34 | How many days were your recent hospitalization? |
|  | Type Number |
| a35 | How much did you pay for your recent hospitalization? |
|  | Type Number |
| a36 | Which of the following reimbursement(s) could you received for your recent hospitalization? |
| a36_1 | Insurance types: basic social insurance, including Urban Resident Basic Medical Insurance (URBMI) and Urban Employee Basic Medical Insurance (UEBMI). (multiple options) |
|  | 1) Yes |
| a36_2 | Insurance types: Shenzhen worker insurance. |
|  | 1) Yes |
| a36_3 | Insurance types: New Cooperative Medical Scheme (NCMS) |
|  | 1) Yes |
| a36_4 | Insurance types: commercial insurance. |
|  | 1) Yes |
| a36_5 | Insurance types: no insurance |
|  | 1) Yes |
| a36_6 | Insurance types: cooperative insurance |
|  | 1) Yes |
| a36_7 | Insurance types: non-adult insurance |
|  | 1) Yes |
| a36_8 | Insurance types: other insurance |
|  | 1) Yes |
| a36_8_text | Insurance types: other insurance text |
|  | Type answer |
|  |  |
| **Part Three: 18-59 Adult survey question** | |
| code | Question |
| b1-b15. Health factors | |
| b1 | Do you smoke? |
|  | 1) Yes; 2) quit; 3) Never |
| b2 | How often do you smoke currently? |
|  | 1) All the time (more than 5 days); 2) Sometimes (in party or business, jump to b4) |
| b3 | How many cigarettes did you smoke past week? |
|  | Type Number |
| b4 | How old were you when you first smoked? Age |
|  | Type Number |
| b5 | If you have quit smoking, how many years have you quitted? Years |
|  | Type Number |
| b6 | In a typical week, how many days do you exposed to secondhand smoke? |
| b6_1 | Not at all |
|  | 1) Yes |
| b6_2 | Yes |
|  | Type Number |
| b7 | Do you drink? |
|  | 1) Yes, I am drinking right now; 2) I have quitted (jump to b9); 3) Never (jump to b11) |
| b8 | Which of the following best describe your current drinking habits? |
|  | 1) 1-2 times a week; 2) 1-2 time a month; 3) rarely |
| b9 | When did you start drinking? Age |
|  | Type Number |
| b10 | If you have quite drinking alcohol, how many years have you quit drinking? Years |
|  | Type Number |
| b11 | In the past month, what is the average accumulated hours spend in sedentary activities (e.g., studying, working, watching TV, using computer, etc.) |
|  | 1) Less than 2 hours per day; 2) 2 to 4 hours per day; 3) 4 to 8 hours per day; 4) 8 to 12 hours per day; and 5) More than 12 hours per day |
| b12 | Within the past six months, what types of recreational physical activity did you participated in? (multiple options) |
| b12_1 | Did not participate in any activities |
|  | 1) Yes |
| b12_2 | Machine equipment physical activity |
|  | 1) Yes |
| b12_3 | Aerobic activity or aerobic dances |
|  | 1) Yes |
| b12_4 | Swimming |
|  | 1) Yes |
| b12_5 | Ambulatory activity (e.g., brisk walking, jogging, running, hiking) |
|  | 1) Yes |
| b12_6 | Ball-related sports (e.g. basketball, baseball, soccer, etc.) |
|  | 1) Yes |
| b12_7 | Sports or fitness competition |
|  | 1) Yes |
| b12_8 | Martial arts |
|  | 1) Yes |
| b12_9 | Other |
|  | 1) Yes |
| b12_9_text | Type the recreational physical activity name |
|  | Type answer |
| b13 | If you were not able to participate in physical activity or exercise each week, the reasons why they were unable to engage in physical activity weekly. (multiple options) |
| b13_1 | No recreational physical activity is needed due to labor intensive occupations |
|  | 1) Yes |
| b13_2 | No time to engage in physical activity |
|  | 1) Yes |
| b13_3 | There were no appropriate places and/or environments for physical activity |
|  | 1) Yes |
| b13_4 | I feel healthy, I do not need physical activity |
|  | 1) Yes |
| b13_5 | Do not want to engage in physical activity |
|  | 1) Yes |
| b13_6 | Feeling ill, unable to participate in physical activity |
|  | 1) Yes |
| b13_7 | Other reasons |
| b13_7_text | Type the reasons |
| b14 | Within the past six months, how often do you exercise per week? |
|  | 1) 6 or more times per week; 2) 3 to 5 times per week; 3) 1 to 2 times per week; and 4) less than 1 time. |
| b15 | On average, how long do you engage in physical activity or exercise in minutes? |
|  | Type Number |
| b16-b29. Dietary status | |
| b16 | How would you describe the home cooked meal at your house? (multiple options) |
| b16_1 | Salty |
|  | 1) Yes |
| b16_2 | Normal |
|  | 1) Yes |
| b16_3 | Light |
|  | 1) Yes |
| b16_4 | Sweeten |
|  | 1) Yes |
| b16_5 | Oily |
|  | 1) Yes |
| b17 | Within the past week, how many days did you have breakfast? |
|  | 1) Less than one day; 2) 1-2 days; 3) 3-4 days; 4) 5-6 days; 5) Everyday |
| b18 | Within the past week, how many days did you eat grains? |
|  | 1) Less than one day; 2) 1-2 days; 3) 3-4 days; 4) 5-6 days; 5) Everyday |
| b19 | Within the past week, how many meals did you eat vegetables in a day? |
|  | 1) Less than one meal/day; 2) One meal/day; 3) Two meals/day; 4) Three meals/day. |
| b20 | Within the past week, how many days did you eat fruits? |
|  | 1) Less than one day; 2) 1-2 days; 3) 3-4 days; 4) 5-6 days; 5) Everyday |
| b21 | In this week, how many days do you eat aquatic product? |
|  | 1) Less than one day; 2) 1-2 days; 3) 3-4 days; 4) 5-6 days; 5) Everyday |
| b22 | Within the past week, how many days did you consume dairy products? |
|  | Type Number |
| b23 | How much ml/g do you drink |
|  | Type Number |
| b24 | When you are thirsty, what do you typically drink? |
|  | 1) Water; 2) Tea/coffee; 3) Soda; 4) Flavor drink; 5) Milk; 6) Juice |
| b25 | Within the past week, how many days did you consumer sugar sweetened carbonated drinks? |
|  | 1) Less than one day; 2) 1-2 days; 3) 3-4 days; 4) 5-6 days; 5) Everyday |
| b26 | Within the past week, how many days did you drink fruit juices? |
|  | 1) Less than one day; 2) 1-2 days; 3) 3-4 days; 4) 5-6 days; 5) Everyday |
| b27 | What are your dietary preferences? |
|  | 1) Vegetables and meat; 2) Meat; 3) Vegetables |
| b28 | Have you heard of the Chinese Pagoda and Dietary guidelines? |
|  | 1) Yes; 2) No (skip to question 30) |
| b29 | What is the characteristic of balance dietary? |
|  | 1) Multiple food but mainly with grain; 2) Vegetables and meat, mainly with grain; 3) Vegetables and meat, mainly with meat; 4) multiple food, mainly with meat; 5) don' t know |
| b30-b33 Self-care status | |
| b30 | When was your most recent weight in? |
|  | 1) Within a month; 2) Within 6 months; 3) Within 12 months; 4) One year ago; 5) Don't know |
| b31 | Within the past 6 months, did you control your weight? |
|  | 1) Yes; 2) No (skip to question 33) |
| b32 | How did you manage your weight? (multiple options) |
| b32_1 | Diet |
|  | 1) Yes |
| b32_2 | Exercise |
|  | 1) Yes |
| b32_3 | Medication use |
|  | 1) Yes |
| b32_4 | Other |
|  | 1) Yes |
| b33 | Where do you typically receive health-related information? (multiple options) |
| b33_1 | From school or working place |
|  | 1) Yes |
| b33_2 | From TV or broadcast |
|  | 1) Yes |
| b33_3 | From blackboard |
|  | 1) Yes |
| b33_4 | From physicians |
|  | 1) Yes |
| b33_5 | From friend's chats or conversation |
|  | 1) Yes |
| b33_6 | From website |
|  | 1) Yes |
| b33_7 | From WeChat, or APPs |
|  | 1) Yes |
| b33_8 | From book, or newspaper |
|  | 1) Yes |
| b33_9 | From individuals who sell supplement products |
|  | 1) Yes |
| b33_10 | Other |
|  | 1) Yes |
| b33_11 | Don't care about |
|  | 1) Yes |
| b34-b60. Basic health knowledge | |
| b34 | What is health to you? |
|  | 1) Physical health; 2) Mental health; 3) Physical and mental health; 4) Physical and mental and social adaptation; 5) Don't know |
| b35 | What do you think is the normal body temperature in Celsius degrees? |
|  | 1) 34-35; 2) 36-37 3) 38-39; 4) Don't know |
| b36 | Do you think that over consumption of salt will affect your health? |
|  | 1) Yes; 2) No; 3) Don't know |
| b37 | When was the last time you measured your blood pressure? |
|  | 1) Within a month; 2) Within three months; 3) Within 6 months; 12) Within 12 months; 5) A year ago; 6) Don't know |
| b38 | Do you think which of the following is the criteria for high blood pressure? |
|  | 1) ≥140/90mmHg; 2) ≥120/80mmHg; 3) ≥165/95mmHg; 4) ≥180/100mmHg; 5) Don't know; 6) Others |
| b39 | Do you know how to treat patients with diagnosed hypertension with medicine? |
|  | 1) Lifetime and consistent taking medicine; 2) When the blood pressure is high; 3) When have the symptoms; 4) Don't know |
| b40 | Which of the following will hypertension cause? (multiple options) |
| b40_1 | Stroke |
|  | 1) Yes |
| b40_2 | Myocardial infarction |
|  | 1) Yes |
| b40_3 | Kidney problem |
|  | 1) Yes |
| b40_4 | Retinopathy |
|  | 1) Yes |
| b40_5 | Don't know |
|  | 1) Yes |
| b40_6 | Other cause |
|  | 1) Yes |
| b40_6_text | Type answer |
| b41 | High blood pressure is association with which of the following? (multiple options) |
| b41_1 | Inherited |
|  | 1) Yes |
| b41_2 | Obesity |
|  | 1) Yes |
| b41_3 | Over salty |
|  | 1) Yes |
| b41_4 | Over drinking |
|  | 1) Yes |
| b41_5 | Smoking |
|  | 1) Yes |
| b41_6 | Nervous |
|  | 1) Yes |
| b41_7 | Lack of exercise |
|  | 1) Yes |
| b41_8 | Deficiency of micronutrients |
|  | 1) Yes |
| b42 | Which of the following fasting blood glucose (FBG) ranges do you think belongs to individuals at high risk for diabetes? |
|  | 1) 5.1≤FBG≤6.0mmol/L; 2) 6.1≤FBG≤7.0mmol/L; 3) 7.0≤FBG≤8.0mmol/L; 4) Don’t know |
| b43 | How should we treat people with diabetes with medicine? |
|  | 1) Lifetime and consistent taking medicine; 2) When the blood sugar is high; 3) When have the symptoms; 4) Don't know |
| b44 | Do you think that smoking and secondhand smoke will affect the development of fetus? |
|  | 1) No; 2) Yes; 3) Don't know |
| b45 | Do you know what kind of methods will transmit HIV? (multiple options) |
| b45_1 | HIV is transmitted through vaginal sex with someone with HIV |
|  | 1) Yes |
| b45_2 | HIV is transmitted through blood |
|  | 1) Yes |
| b45_3 | HIV is transmitted through birth |
|  | 1) Yes |
| b45_4 | HIV is transmitted by everyday engagement with someone with HIV |
|  | 1) Yes |
| b45_5 | HIV is transmitted through air |
|  | 1) Yes |
| b45_6 | Don’t know |
|  | 1) Yes |
| b46 | How do you think Hepatitis B is transmitted? |
|  | 1) Working and eating; 2) Sex, blood, and pregnant 3) Handshaking and huge; 4) Don't know |
| b47 | What measure do you think individuals with chronic disease/condition should take? (multiple options) |
| b47_1 | Visit the physician in time |
|  | 1) Yes |
| b47_2 | Standardized and consistent treatment |
|  | 1) Yes |
| b47_3 | Rational use of medicines |
|  | 1) Yes |
| b47_4 | Prevent complications |
|  | 1) Yes |
| b47_5 | Improve the quality of life |
|  | 1) Yes |
| b47_6 | Don't know |
|  | 1) Yes |
| b48 | What do you think included in healthy lifestyle? (multiple options) |
| b48_1 | Balance diet |
|  | 1) Yes |
| b48_2 | Keep mentally health |
|  | 1) Yes |
| b48_3 | Quit smoking, and limited drinking |
|  | 1) Yes |
| b48_4 | Exercise properly |
|  | 1) Yes |
| b48_5 | Keep enough sleep |
|  | 1) Yes |
| b48_6 | Pay attention to personal hygiene |
|  | 1) Yes |
| b48_7 | Don't know |
| b49 | What is the healthy intake of vegetables? |
|  | 1) 300-500g every day, blank vegetables need to be 1/2; 2) 300-500g every day, dark vegetables need to be 1/2; 3) 100-300g every day, blank vegetables need to be 1/2; 4) 100-300g every day, dark vegetables need to be 1/2; 5) Don't know |
| b50 | How much milk do you think adults should drink? |
|  | 1) 150g; 2) 300g; 3) 500g; 4) Don't need to drink every day |
| b51 | Which of the following is incorrection about fish, chicken, meat, and eggs? |
| b51_1 | Choose fish and chicken first |
|  | 1) Yes |
| b51_2 | Eat egg without yolk |
|  | 1) Yes |
| b51_3 | Don't need to limit the smoky meat intake |
|  | 1) Yes |
| b51_4 | Only choose to eat lean from the meat |
|  | 1) Yes |
| b52 | How much water do you think you need to drink each day? |
|  | 1) 3-4 cups (600ml to 800ml); 2) 5-6 cups (1000ml to 1200ml); 3. 7-8 cups (1500ml to 1700ml); 4) Don't know |
| b53 | What is the limit intake of salt each day for adults? |
|  | 1) 3g; 2) 6g; 3) 10g; 4) Don't know |
| b54 | What is the limit intake for cooking oil each day for adults? |
|  | 1) 10-20g; 2) 25-30g; 3) 40-50g; 4) Don't know |
| b55 | Which of the following types of fats are harmful to the body? |
|  | 1) Polyunsaturated fatty acid 2) Monounsaturated fatty acid; 3) Trans-fatty acid; 4) Don't know |
| b56 | Do you have the habits of reading the nutrition panel on the food label? |
|  | 1) Never (jump to b58); 2) Sometimes; 3) All the time; 4) Every time |
| b57 | When reading the nutrition panel of the food label, what should we focus on? (multiple options) |
| b57_1 | Protein |
|  | 1) Yes |
| b57_2 | Sugar |
|  | 1) Yes |
| b57_3 | Trans-fatty acid |
|  | 1) Yes |
| b57_4 | Sodium |
|  | 1) Yes |
| b58 | Do you know the recommended intake of the following type of food each day and each week? |
|  | 1) 12 and 25; 2) 5,10; 3) Don't know |
| b59 | Do you know the BMI ranges for normal wight? |
|  | 1)18.5≤BMI＜24.0; 2)19.5≤BMI＜25.0; 3) Don't know |
| b60 | Do you know the formula for calculating BMI? |
|  | 1) BMI=hight (m)^2/ weight (kg); 2) BMI=weight(kg)/hight (m)^2 3) Don't know |
| b61 - b79. Community health care center awareness and utilization | |
| b61 | Which medical institutions do you frequently visit? |
|  | 1) Private clinic; 2) Community health care center; 3) District level general hospital; 4) City level general hospital; 5) District level or city level special hospital; 6) Province level hospital; 7) others |
| b62 | The reason that you choose this medical institution. (multiple choose) |
| b62_1 | Proximity to the home |
|  | 1) Yes |
| b62_2 | Reasonable cost |
|  | 1) Yes |
| b62_3 | Skilled personnel |
|  | 1) Yes |
| b62_4 | Quality medical equipment |
|  | 1) Yes |
| b62_5 | Abundant medicine types |
|  | 1) Yes |
| b62_6 | Excellent customer services |
|  | 1) Yes |
| b62_7 | Assigned relationship to the health care institutions |
|  | 1) Yes |
| b62_8 | Familiarity with personnel |
|  | 1) Yes |
| b62_9 | Trust in certain physicians |
|  | 1) Yes |
| b62_10 | Other |
|  | 1) Yes |
| b62_11_text | Type Number |
| b63 | Do you know where is the nearest CHCs from your home? How long does it take you to go to the nearest community healthcare center from your home? |
| b63_text | Type number |
| b64 | What is your purpose of visiting CHCs? (multiple choose) |
| b64_1 | Visit the physician |
|  | 1) Yes |
| b64_2 | Prescribe medicine |
|  | 1) Yes |
| b64_3 | Chronic disease following up |
|  | 1) Yes |
| b64_4 | Consultant |
|  | 1) Yes |
| b64_5 | Acupuncture |
|  | 1) Yes |
| b64_6 | Vaccination |
|  | 1) Yes |
| b64_7 | Children physical exam |
|  | 1) Yes |
| b64_8 | Other |
| b64_8_text | Type answer |
| b65 | Does the doctors at the community healthcare center discuss with you regarding community health? |
|  | 1) Yes; 2) No |
| b66 | Have you ever heard about family doctor contract services? (If the answer is "No", jump to b73 |
|  | 1) Yes; 2) No |
| b67 | Where do you heard about the family doctor contract services? (multiple choose) |
| b67_1 | Community health care personnel/providers |
|  | 1) Yes |
| b67_2 | Broadcast, TV |
|  | 1) Yes |
| b67_3 | Newspaper |
|  | 1) Yes |
| b67_4 | Website |
|  | 1) Yes |
| b67_5 | Other |
|  | 1) Yes |
| b67_6 | Don't know |
| b68 | Did you sign a service contract with a family doctor? |
|  | 1) Yes; 2) No; 3) Don't know (if the answer is "No" or "Don't know", jump to a73) |
| b69 | Do you think the family doctor contract service is beneficial for disease prevention? |
|  | 1) Very helpful; 2) Helpful; 3) Normal; 4) Not helpful; 5) Helpless |
| b70 | After signing a service contract with a family doctor, are you more likely to visit the healthcare institutions where the family doctor located? |
|  | 1) Yes; 2) No |
| b71 | Do you think that the community health center provides outpatient appointment to individuals whom sign a service contract with a family doctor? |
|  | 1) Yes; 2) No |
| b72 | Which methods do you want to the most when conducting health consultation with family doctor? (multiple choose) |
| b72_1 | Go to the medical providers |
|  | 1) Yes |
| b72_2 | Telephone consultant |
|  | 1) Yes |
| b72_3 | Email |
|  | 1) Yes |
| b72_4 | Community professional app |
|  | 1) Yes |
| b72_5 | Text |
|  | 1) Yes |
| b72_6 | QQ or WeChat |
|  | 1) Yes |
| b73 | In the last year, have you participated the health conference in the center? |
|  | 1) Yes; 2) No |
| b74 | Within the past year, did you see any community health care center advisements? |
|  | 1) Yes; 2) No |
| b75 | Did you know that community health care center provided chronic disease management services? |
|  | 1) Yes; 2) No |
| b76 | Are you satisfied with the environment of the CHC? |
|  | 1) Very satisfied; 2) Satisfied; 3) Not so bad; 4) Dissatisfied; 5) Very dissatisfied; 6) Don't know |
| b77 | Are you satisfied with the attitude of health care workers? |
|  | 1) Very satisfied; 2) Satisfied; 3) Not so bad; 4) Dissatisfied; 5) Very dissatisfied; 6) Don't know |
| b78 | Are you satisfied with the communication? |
|  | 1) Very satisfied; 2) Satisfied; 3) Not so bad; 4) Dissatisfied; 5) Very dissatisfied; 6) Don't know |
| b79 | Are you satisfied with the (medical) skills of health workers |
|  | 1) Very satisfied; 2) Satisfied; 3) Not so bad; 4) Dissatisfied; 5) Very dissatisfied; 6) Don't know |
| b80-89. Mental health | |
| b80 | In the past 30 days, how often do you feel nervousness, anxious, and irritated? |
|  | 1) All the time; 2) Most of the time; 3) Sometimes; 4) None |
| b81 | In the past 30 days, how often do you feel hopelessness, depressed, bored, and felt nothing can make you happy? |
|  | 1) All the time; 2) Most of the time; 3) Sometimes; 4) None |
| b82 | In the past 30 days, how often do you feel useless and everything is too hard? |
|  | 1) All the time; 2) Most of the time; 3) Sometimes; 4) None |
| b83 | If you are going to travel, would it be easy for you to find other to join you? |
|  | 1) All the time; 2) Most of the time; 3) Sometimes; 4) None |
| b84 | How often do you talk to your friends and family members about your problems and fears? |
|  | 1) All the time; 2) Most of the time; 3) Sometimes; 4) None |
| b85 | If you are sick, how often can you have someone help you with errands and housework? |
|  | 1) All the time; 2) Most of the time; 3) Sometimes; 4) None |
| b86 | If you decided to watch a movie, how often can you find someone to watch it with you easily? |
|  | 1) All the time; 2) Most of the time; 3) Sometimes; 4) None |
| b87 | How often do you invited other people to join you for various activities (e.g., shopping, eating, watching movies, or non-job-related activities)? |
|  | 1) All the time; 2) Most of the time; 3) Sometimes; 4) None |
| b88 | If you want to find someone to join you for lunch, how often can you find someone easily? |
|  | 1) All the time; 2) Most of the time; 3) Sometimes; 4) None |
| b89 | If you encounter difficulties, how often can you call someone to help you easily? |
| b90-b98. First aid knowledge | |
| b90 | Do you think community residents should learn about emergency related knowledge? |
|  | 1) Totally agree; 2) Agree; 3) Not important; 4) Don't know |
| b91 | When there is an accident, what is your first reaction? |
|  | 1) Don’t know what to do; 2. Call 119 and wait for help; 3) Call 119 and do some first aid before the ambulance come; 4) First aid immediately |
| b92 | Have you heard of CPR? (If answer is no, jump to b95) |
|  | 1) Yes; 2) No |
| b93 | CPR is an important lifesaving technique; do you know what is the first step of CPR? |
|  | 1) Artificial breathing; 2) Open breath pattern; 3) Circulation |
| b94 | How many minutes after the heart after beating is the golden stage for first aid? |
|  | 1) 2-4; 2) 4-6; 3) 6-8; 4) 8-10 |
| b95 | Which of the following is the treatments to heat stroke? (multiple choose) |
| b95_1 | Drink how water or light salty water |
|  | 1) Yes |
| b95_2 | Drink warm water |
|  | 1) Yes |
| b95_3 | Pinch people's philtrum |
|  | 1) Yes |
| b95_4 | CPR |
|  | 1) Yes |
| b96 | Which of the following is the correct treatment for burn? |
|  | 1) Wash by cold water for 10-15 min; 2) Apply toothpaste, soy sauce, etc. on the wound; 3) Tie tightly with sterile gauze or cotton cloth |
| b97 | Is there a fire extinguisher in your work and living environment? |
|  | 1) Yes; 2) No |
| b98 | Do you know how to correctly use a fire extinguisher? |
|  | 1) Yes; 2) No |
